# microRNA-194 suppresses osteosarcoma cell proliferation and metastasis *in vitro* and *in vivo* by targeting CDH2 and IGF1R

**DOI:** 10.3892/ijo.2014.2571

**Published:** 2014-07-30

**Authors:** KANG HAN, TINGBAO ZHAO, XIANG CHEN, NA BIAN, TONGTAO YANG, QIONG MA, CHENGKUI CAI, QINGYU FAN, YONG ZHOU, BAOAN MA

**Affiliations:** 1 Department of Orthopedic Surgery, Orthopedics Oncology Institute of Chinese PLA, Tangdu Hospital, Fourth Military Medical University, Xi’an, Shaanxi, P.R. China; 2 Department of Spinal Cord Injury, General Hospital of Jinan Military Area Command of Chinese PLA, Jinan, Shandong, P.R. China; 3 Department of Baylor College of Medicine, Houston, TX, USA

**Keywords:** osteosarcoma, miR-194, CDH2, IGF1R, metastasis, proliferation

## Abstract

Studies have shown that miR-194 functions as a tumor suppressor and is associated with tumor growth and metastasis. We studied the effects of miR-194 in osteosarcoma and the possible mechanism by which miR-194 affected the survival, apoptosis and metastasis of osteosarcoma. Both human osteosarcoma cell lines SOSP-9607 and U2-OS were transfected with recombinant lentiviruses to regulate miR-194 expression. Overexpression of miR-194 partially inhibited the proliferation, migration, and invasion of osteosarcoma cells *in vitro*, as well as tumor growth and pulmonary metastasis of osteosarcoma cells *in vivo*. Potential miR-194 target genes were predicted using bioinformatics. Luciferase reporter assay, real-time quantitative PCR and western blotting confirmed that *CDH2 (N-cadherin)* and *IGF1R* were targets of miR-194. Using real-time quantitative PCR, we evaluated the expression of miR-194 and two miR-194 target genes, *CDH2* and *IGF1R* in osteosarcoma samples from 107 patients and 99 formalin- or paraformalin-fixed paraffin-embedded tissues. The expressions of the target genes were also examined in osteosarcoma samples using immunohistochemistry. Overexpression of miR-194 inhibited tumor growth and metastasis of osteosarcoma probably by downregulating *CDH2* and *IGF1R*. miR-194 may prove to be a promising therapeutic agent for osteosarcoma.

## Introduction

Osteosarcoma (OS) is the most common primary malignant neoplasm in adolescents with an annual estimated worldwide incidence of 4 million, with a peak incidence at the age of 15–19 years (1). Osteosarcoma is associated with abnormal differentiation caused by genetic and epigenetic changes. Advances in osteosarcoma therapy have enhanced patient outcomes. The most effective regimens currently include neoadjuvant and adjuvant chemotherapy coupled with local control that usually consists of limb-sparing surgery (2). Unfortunately, the cure rate is still very poor due to pulmonary metastases (3). Therefore, the identification of effector molecules and signaling pathways underlying resistance to chemotherapy and malignancy is vital for osteosarcoma treatment. Studies have investigated the genes associated with metastasis of osteosarcoma, and microRNAs (miRNAs) have become a new research hotspot in gene therapy.

microRNAs (miRNAs) are a class of 22–25 nucleotide RNA molecules that negatively regulate gene expression in animals and plants (4,5). Since the discovery of the role of miRNAs in *Caenorhabditis elegans* development (6), a frequent disregulation of miRNAs has been observed in diverse cancers, including synovial sarcoma, colon cancer (7), breast cancer (8), glioma (9), glioblastoma (10), hepatocellular carcinoma (11), lung (12) and gastric cancer (13). Some of these miRNA expression profiles showed downregulation in tumors compared with normal tissue (14), like miR-127 in human bladder cancers (15) and microRNA-34a in OS (16). However, other miRNAs are upregulated in tumors, such as miR-150 in gastric cancer (17) and miR-17-92 cluster in renal cell carcinoma (18). The alterations in miRNA expression may play a crucial role in the initiation and progression of the above cancers (19), functioning as a novel class of oncogenes and tumor suppressors (20,21). Thus, miRNAs play an essential role in basic physiologic processes, such as development, differentiation, proliferation and apoptosis (22). However, their biological function remains largely unknown.

miR-194 is specifically expressed in the human gastrointestinal tract and is induced during intestinal epithelial cell differentiation (23). The regulatory role of miR-194 was first studied in normal and malignant cells of the gastrointestinal tract (24). Overexpression of miR-194 in gastrointestinal cancer cells suppresses cell migration, invasion and metastasis (24). miR-194 functions as a tumor suppressor gene by downregulating targets such as *SSH2*, *HBEGF*, *IGF1R*, *CDH2 (N-cadherin)* and *TLN2* (23–27). Hepatocyte nuclear factor (HNF) also induces miR-194 expression during intestinal epithelial cell differentiation (23). In colon cancer tissue, miR-194 was downregulated relative to normal mucosa (28). Low expression of miR-194 has been associated with large tumor size and advanced stage in gastric cancer (29). In endometrial cancer cells, miR-194 has been reported to inhibit self-renewal factor BMI-1, reduce cell invasion and inhibit epithelial-mesenchymal transition (EMT) (30). The mutations of p53 tumor suppressor gene, which directly regulates the expression of miR-194, were found in 20–60% of sporadic OS (31). The reports suggested that miR-194 may function as a tumor suppressor in OS. However, the effects of miR-194 in osteosarcoma have not been completely elucidated. Therefore, it is of great significance to further study the function and mechanism of miR-194 in osteosarcoma.

We carried out *in vitro* and *in vivo* experiments to evaluate the effects of miR-194 and its possible direct targets, *IGF1R* and *CDH2*, in tumor growth and metastasis of SOSP-9607 and U2-OS cells. We also predicted its putative target genes, which are correlated with tumor growth and metastasis, using bioinformatics analysis. We report for the first time that overexpression of miR-194 inhibited growth and metastasis of osteosarcoma. In addition, miR-194 specifically downregulated the expression of *IGF1R* and *CDH2*. miR-194 gene therapy may prove to be a promising therapy for tumorsuppression in osteosarcoma.

## Materials and methods

### Ethics statement

All research involving human tissue samples and animals was approved by the Ethics Review Committee of Fourth Military Medical University, Xi’an, Shaanxi, China (approval ID:2013106) and written informed consent was obtained from all participating patients.

### Human tissue samples

A total of 107 pairs of human osteosarcoma tissue samples were obtained from patients who underwent surgical resection at the Tangdu Hospital of Fourth Military Medical University between 2007 and 2010 and were diagnosed with osteosarcoma based on histopathological evaluation. The biopsies were immediately snap-frozen in liquid nitrogen after resection and stored at -80°C. One section of each sample was stained with hematoxylin-eosin (H&E) for histopathological evaluation. The clinical stage of these osteosarcoma patients was classified according to the sixth edition of the tumor-node-metastases (TNM) classification of the International Union Against Cancer (UICC).

All 107 osteosarcoma patients were studied in a follow-up. The median follow-up was 42 months (range, 5–68 months). During the follow-up period, 62 patients (57.9%) died of disease. Distant metastases developed in 52 patients at a mean of 12.7 months (range, 3–41 months) after the original diagnosis. Of these patients, 13 had bone metastases and 43 had lung metastases (4 patients had both bone and lung metastases).

### Cell culture

Human osteosarcoma cell lines SOSP-9607 were established and reserved in our laboratory as previously described (32). Human osteosarcoma cell lines U2-OS were purchased from the American Type Culture Collection (ATCC, Manassas, VA, USA) and used within 6 months of the purchase. SOSP-9607 cells were maintained in RPMI-1640 medium (HyClone, Logan, UT, USA) supplemented with 10% fetal bovine serum (FBS; HyClone), 2.0 mM l-glutamine, 100 U/ml penicillin and 100 μg/ml streptomycin, and incubated at 37°C in a humidified incubator supplemented with 5% CO_2_ and 95% air. U2-OS cells were maintained in the same conditions, except DMEM medium was used. Cell line authentication was performed by the Orthopedics Oncology Institute of Chinese PLA according to the UKCCCR guidelines every 2–3 months, including mycoplasma test by PCR and measurement of cell proliferation by counting.

### Generation of stable cell lines

Recombinant lentiviruses containing overexpression of miRNA-194, for knocking down miRNA-194 and miRNA control were purchased from Shanghai Genechem Co., Ltd. (Shanghai, China). The precusor sequence of miR-194 was used for overexpression as follows: AUGGUGUUAUCAAGUGUAACAGCAACUCCAUGUGG ACUGUGUACCAAUUUCCAGUGGAGAUGCUGUUACU UUUGAUGGUUACCAA. The reverse complementary sequence of miR-194 was used for the knock-down as follows: TC CACATGGAGTTGCTGTTACA. Besides the multiple clone sites of lentivirus expression vectors, there also was a GFP reporter driven by an independent promoter (SV40 promoter) to indicate the infection rate of the virus timely.

To generate the stable cell line, 1×10^4^ cells were transfected with 5×10^5^ transducing units of lentiviruses. The supernatant was removed after 24 h and replaced with complete culture medium. Infection efficiency was confirmed by RT-PCR 96 h after infection and the cells were selected with 1 μg/ml puromycin for 2 weeks.

### Reverse transcription and quantitative real-time PCR

Total RNA containing miRNA and mRNA was extracted from cells with TRIzol^®^ reagent (Invitrogen, Carlsbad, CA, USA), or from formalin- or paraformalin-fixed, paraffin-embedded (FFPE) tissues with RecoverAll™ Total Nucleic Acid Isolation kit (Ambion, Foster City, CA, USA; cat no. AM1975), according to the manufacturer’s instructions. The RNA was quantified by absorbance at 260 nm and transcribed into cDNA using BioRT Two-Step RT-PCR kit (Bioer Technology, Inc., Hangzhou, China). To evaluate IGF-1R and N-cadherin expression levels, 1 μg of total RNA was used for reverse transcription with iScript cDNA Synthesis kit (Bio-Rad Laboratories, Hercules, CA, USA), according to the manufacturer’s instructions. The sequences of the forward and reverse primers for IGF1R were 5′-CGACATTGAGGAGGTCAC AGA-3′ and 5′-TGGGCACGAAGATGGAGTT-3′. The sequences of the forward and reverse primers for N-cadherin were 5′-GTCAGCAGAAGTTGAAGAAATAGTG-3′ and 5′-GCAAGTTGATTGGAGGGATG-3′.

The sequences of the forward and reverse primers for glyceraldehyde-3-phosphate dehydrogenase (GAPDH) were 5′-TGGGTGTGAACCATGAGAAGT-3′ and 5′-TGAGTCC TTCCACGATACCAA-3′. The expression level of GAPDH was used as a control. To evaluate hsa-mir-194 levels, the sequences of primers for miR-194: 5′-ACACTCCAGCTGGG TGTAACAGCAACTCCAT-3′ were used. U6 was used as a control.

### Apoptosis, proliferation and cell cycle assays

Cultured cells were grown in 6-, 24- and 96-well plates. Apoptosis and cell cycle were measured using flow cytometry. The procedures were performed as previously described (33). Cell viability was examined using the 3-(4,5-dimethylthiazol-2-yl)-2,5-diphenyltetrazolium bromide (MTT) assay. MTT was performed at 24, 48, 72, 96, 120 and 144 h. The absorbance at 492 nm was measured after incubation with 20 μl of MTT for 4 h. The curve of cell proliferation was then drawn and the proliferation efficiency was examined. There were 6-wells in each group, the experiments were repeated three times independently and the results were given as means ± SD. The plate clone formation assay was performed as previously described (34). Clones with >50 cells were counted with an ordinary optical microscope and the clone formation rate was calculated with the following formula: Plate clone formation efficiency = (number of clones/number of cells inoculated) × 100%.

### Transwell cell migration and matrigel invasion assays

The invasive potential of cells was measured in 6.5 mm Transwell with 8.0-mm pore polycarbonate membrane insert (cat. 3422; Corning, NY), according to the manufacturer’s instructions. The filter of the top chamber was coated with 50 μl of diluted matrigel and incubated at 37°C for 2 h. The lower chambers were filled with 600 μl of RPMI-1640 medium containing 5% fetal bovine serum (FBS) as chemoattractant. The suspension of 5,000 cells in 100 μl migration medium was added into each top chamber. After the cells were incubated for 16 h, the non-invading cells that remained on the upper surface were removed with a cotton swab. The invasive cells in the lower surface of the membrane insert were fixed with 4% paraformaldehyde for 30 min, permeabilized with 0.2% Triton X-100 at room temperature for 15 min and then stained with 0.1% crystal violet for 5 min. The number of cells in the lower surface, which had invaded through the membrane, was counted under a light microscope in five random fields at a magnification of ×100. The experiments were repeated three times independently and results were expressed as means ± SD.

The procedure for transwell migration assays was the same as the transwell invasion assay except that the filter of top chamber was not coated with matrigel.

### Wound healing migration assay

When the transfected and untransfected SOSP-9607 and U2-OS cells were grown to confluence, a scratch in the cell monolayer was made using a micropipette tip. Following incubation of the cells under standard conditions for 24 h, the plates were washed twice with fresh medium and images were captured at different times. The migration potential was estimated by counting the cells that migrated from the wound edge. The cell migration rate was obtained by counting three fields per area and represented as the average of six independent experiments over multiple days.

### Animal studies

Four-week-old female nude mice (BALB/c, nu/nu; Experimental Animal Centre of the Fourth Military Medical University in China), 17–22 g in weight, were maintained under specific pathogen-free conditions with 12-h light/12-h dark cycles at 26–28°C and 50–65% humidity. Animal feed and underpad, which were purchased from the Experimental Animal Center, Fourth Military Medical University, were autoclaved and vacuum packed. The water was adjusted to a pH value of 2.8 and autoclaved before use.

Animal experiments were performed to evaluate orthotopic tumor growth and spontaneous pulmonary metastasis properties of osteosarcoma cells *in vivo*. Briefly, 4 groups SOSP-9607 cells (overexpression of miRNA-194, for knocking down miRNA-194, miRNA control and untransfected cells) suspension of 100,000 cells in 100 μl were injected into the proximal tibia of each anesthetized nude mouse (n=10 animals per group). Every 7 days post-inoculation, the length and width of individual orthotopic tumor were measured with calipers, and the volume (mm^3^) was calculated according to the formula: 1/2 × length × width^2^ (35). The curve of orthotopic tumor growth was depicted 42 days after inoculation mouse lungs and orthotopic tumors were harvested and weighed. miR-194 expression levels in the orthotopic tumors were tested using real-time RT-PCR, and the number of pulmonary metastatic tumor nodules was counted under a low-power dissecting stereomicroscope. Finally, mouse lungs were fixed with 10% neutral-buffered formalin, embedded in paraffin, sectioned at 6 μm and stained with H&E. The pulmonary metastases were imaged under a light microscope at magnifications of ×40, ×100, 2×00 and ×400.

### Protein extraction and western blot analysis

Protein extracts were prepared through a modified RIPA buffer with 0.5% sodium dodecyl sulfate (SDS) in the presence of a proteinase inhibitor cocktail (Complete Mini; Roche Diagnostics, Basel, Switzerland) and then were performed as previously described (36).

### Luciferase reporter assay

To validate *IGF1R* and *N-cadherin* as target genes of miR-194 in osteosarcoma cells, luciferase assay was performed as previously described (33).

### Target prediction

Bioinformatics analysis was carried out using specific programs: Pictar (http://pictar.mdc-berlin.de/), miRanda (http://www.microrna.org) and TargetScan (http://www.targetscan.org/).

### Immunohistochemistry

The dilution of CDH2 and IGF1R antibody used for immunohistochemistry was 1:100. Immunohistochemistry was carried out as previously described (37). The final scores of CDH2 and IGF1R expression were calculated as previously described (38) and classified as follows: 0–4, low; 5–9, high.

### Statistical analysis

All values in the present study were expressed as the means ± SD, and all error bars represent the standard deviation of the mean. Student’s t test, one-way analysis of variance and repeated measures data of ANOVA were used to determine significance. Patient survival and their differences were determined using the log-rank test. Cox regression (proportional hazards model) was used for multivariate analysis of prognostic factors. All statistical tests were two-sided. p<0.05 was considered statistically significant. Statistical analyses were performed using the SPSS 17.0 software (SPSS, Inc., Chicago, IL, USA).

## Results

### Significant difference between F4 and control F5M2 cells

**Figure 1:**
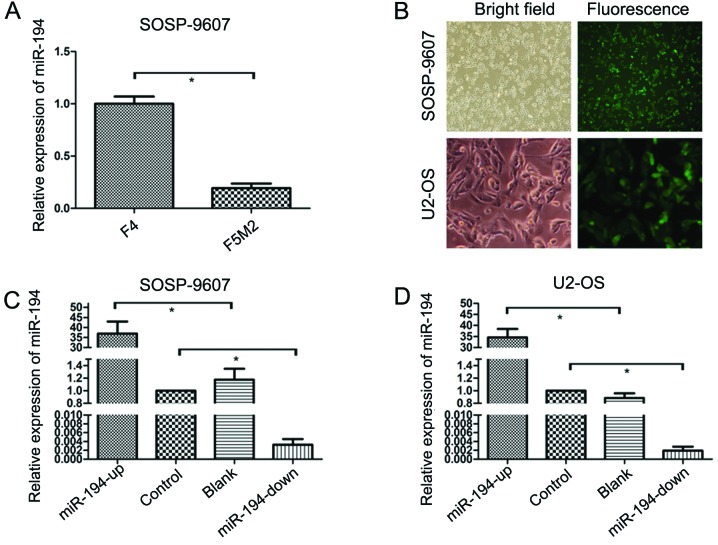
miR-194 expression and miR-194 oligonucleotide transfection in osteosarcoma cells. (A) The qRT-PCR of miR-194 expression in F4 and F5M2 cells (p=0.001). (B) SOSP-9607 cells (top) and U2-OS cells (bottom) were observed with white bright (left) and green fluorescence assay (right) in the same view using fluorescence microscopy (magnification, ×100, ×400). (C) qRT-PCR analysis of miR-194 expression in transfected SOSP-9607 cells. (D) miR-194 expression was assayed in transfected U2-OS cells. U6 was used as an internal loading control. (C and D), data are presented as means ± SD. Columns, mean of three independent experiments; bars, SD; ^*^P<0.05.

F4 and F5M2 were the sublines originated from SOSP-9607 using limited dilution method (32,39). F5M2 cells show stronger proliferation and invasion than F4 cells, which is useful in studies on metastatic mechanism of osteosarcoma (32). In the present study, we evaluated the expression of miR-194 using quantitative real-time PCR. The results showed that miR-194 expression was significantly lower in F5M2 cells compared with F4 cells (Fig. 1A; p<0.001). The results suggested that miR-194 might play a vital role in the metastatic processes.

### Generation of stable cell lines

After transfection and selection of cells, the experiments with SOSP-9607 cells and U2-OS cells were divided into four groups including a blank group (untransfected cells), a control group (cells transfected with the control lentivirus), an OE group (overexpression of miRNA-194) and a KD group (knocked down miRNA-194). These GFP-labeled oligonucleotides were detected using fluorescence microscopy (Olympus, Tokyo, Japan) (Fig. 1B).

miR-194 expression levels in four groups were measured using microscopy and stem-loop real-time RT-PCR. The results (Fig. 1C and D) showed that the level of miR-194 was significantly higher in the OE group and lower in KD group compared with control and blank groups, respectively. However, there were no significant differences between the control and blank groups. These results indicated that miR-194 recombinant lentiviruses could regulate miR-194 expression effectively in both SOSP-9607 and U2-OS cells. These strategies were then used as the basis of the remaining experiments.

### miR-194 inhibits proliferation of osteosarcoma in vitro

**Figure 2:**
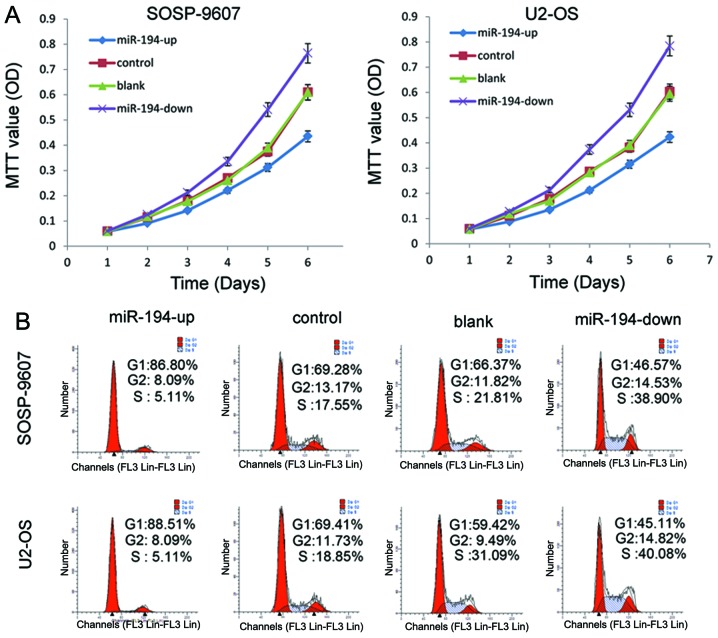
miR-194 inhibits cell proliferation in both SOSP-9607 and U2-OS cell lines. (A) MTT assay of four groups of SOSP-9607 and U2-OS cells. The viable cell number was evaluated as the absorbance at 490 nm with a reference wavelength of 630 nm. Values of optical density (OD) are expressed as means ± SD. Point symbol, mean of four independent experiments; bars, SD. (B) Cell cycle distribution of treated SOSP-9607 (top) and U2-OS (bottom) was measured using flow cytometric analysis.

The results of MTT assay showed that SOSP-9607 cells in OE groups exhibited a significant decline in proliferation capacity compared with the other three groups (p<0.001), which were negatively correlated with the exogenous miR-194 level (Fig. 2A). In contrast, cells in the KD group showed significantly enhanced proliferation (p<0.001). No statistical difference was found between the blank groups and control groups (p=0.541). We also tested U2-OS cells (Fig. 2A). The results were similar to the stably transfected SOSP-9607 cells.

Cell cycle distribution was detected by flow cytometry. The results showed that more cells were in the G0/G1 phase in the OE group compared with the G0/G1 phase in KD group in SOSP-9607 and U2-OS cells (Fig. 2B). These data demonstrated that miR-194 could inhibit the proliferation in both SOSP-9607 and U2-OS cells.

### miR-194 induces apoptosis

**Figure 3:**
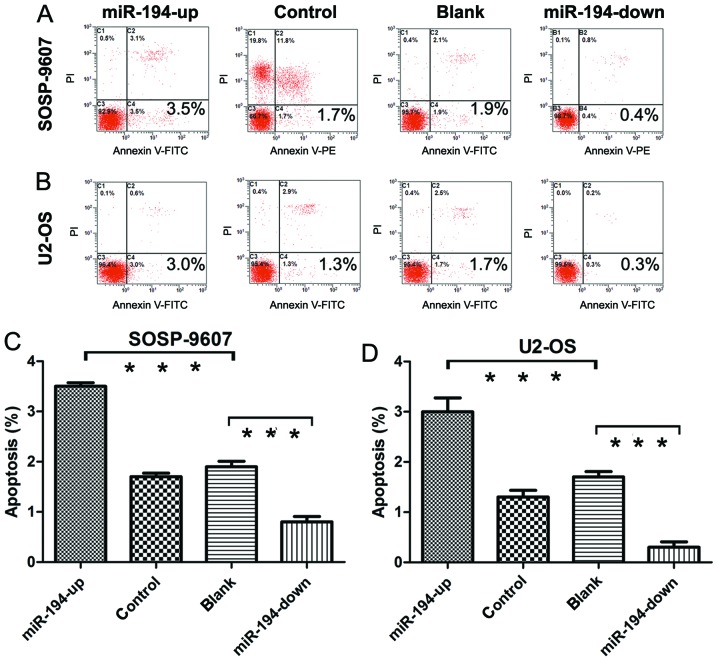
miR-194 induces apoptosis in both SOSP-9607 and U2-OS cells. (A and C) Apoptosis of SOSP-9607 cells were measured using FACS with Annexin V and propidium iodide (PI) staining. (B and D) Apoptosis of U2-OS was measured by FACS with Annexin V and propidium iodide staining. The data are presented as the means ± SD in C and D. Columns, mean of three independent experiments; bars, SD; ^***^P<0.001.

Apoptosis in the SOSP-9607 cell line was detected using flow cytometry. SOSP-9607 cells in the upregulated groups showed significantly increased spontaneous apoptosis compared with the other three groups (p<0.001), with the cells in the downregulated groups showing significantly decreased spontaneous apoptosis. The cells in untransfected groups did not produce noticeable changes compared with cells in the control groups (p=0.147); (Fig. 3A and C). Similar results were obtained in U2-OS cell lines (Fig. 3B and D). These results showed that miR-194 could induce apoptosis in both SOSP-9607 and U2-OS cells.

### Effect of miR-194 on clone formation in SOSP-9607 and U2-OS cells

**Figure 4:**
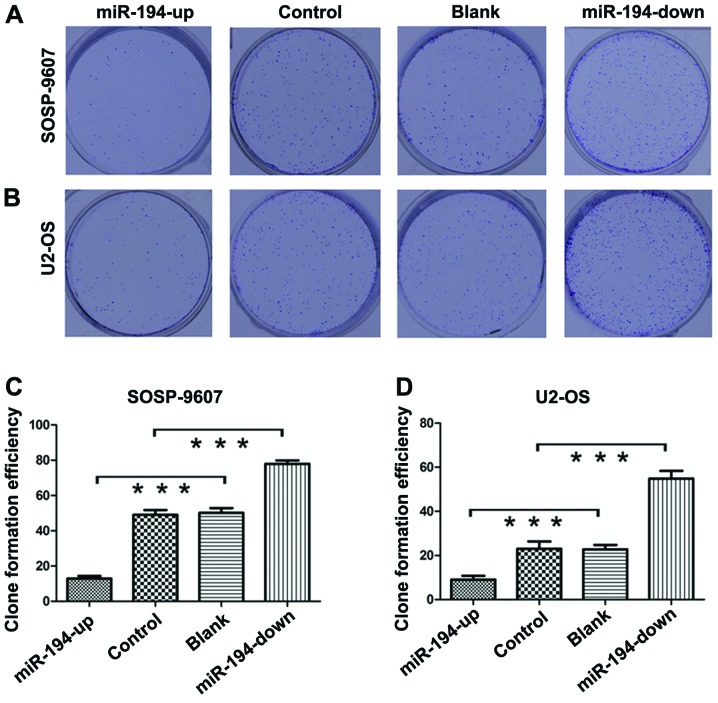
Effect of miR-194 on clonogenicity in both SOSP-9607 and U2-OS cells. (A and C) The clonogenicity of SOSP-9607 cells was captured and counted after 21 days of culture. (B and D) The clonogenicity of U2-OS cells was captured and counted after 21 days of culture; columns, mean of three independent experiments; bars, SD; ^***^P<0.001.

The clone formation efficiency of SOSP-9607 cells was as follows: OE (12.87±2.66%), control (49.00±4.80%), blank (50.13±4.71%) and KD (77.93±3.30%) groups, respectively, after 21 days of culture (Fig. 4A and C). No significant differences existed between the blank and control SOSP-9607 cells (p=0.736). The clone formation efficiency of U2-OS cells was: OE (9.07±2.93%), control (23.00±5.79%), blank (22.8±3.41%) and KD (54.87±6.07%) groups, respectively (Fig. 4B and D). No significant difference was observed between the blank and control cells (p=0.96). Statistical analysis showed that miR-194 inhibits the clonogenicity of osteosarcoma *in vitro (*P<0.001).

### miR-194 inhibits migration and invasion of osteosarcoma in vitro

**Figure 5:**
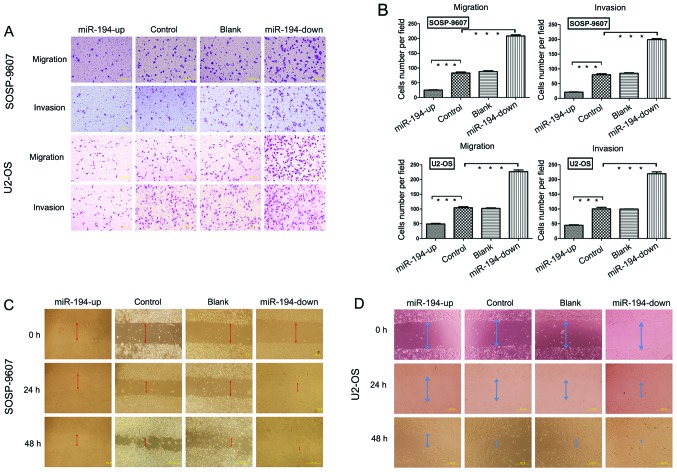
miR-194 inhibits migration and invasion of osteosarcoma *in vitro*. (A) Representative images of migrated and invaded SOSP-9607 or U2-OS cells on the membrane at a magnification of ×200. (B) Quantitative results of the migration and invasion in each group of SOSP-9607 or U2-OS cells are presented, 16 h after incubation. (C and D) SOSP-9607 or U2-OS cells were seeded in 6-well plates and subjected to wounding on the next day. Images were taken at 0, 24 and 48 h, respectively, after the wound. Data are expressed as means ± SD; Columns, mean of three independent experiments; bars, SD; ^***^P<0.001.

Results in the transwell migration assay showed significantly lower numbers of OE SOSP-9607 cells (25.00±2.54; p<0.001) compared with blank (88.00±6.59), control (83.00±7.51) and KD SOSP-9607 cells (208.60±9.04) (Fig. 5A and B). No significant difference was seen between the blank and control SOSP-9607 cells (p=0.266). In the invasion assay, the OE SOSP-9607 cells (21.00±2.23; p<0.001) passing through the matrigel were significantly lower than the blank (80.00±8.30), control (85.00±4.12) and KD SOSP-9607 cells (199.20±7.72) (Fig. 5A and B). No significant difference existed between the blank and control SOSP-9607 cells (p=0.216) (Fig. 5B). Similar results were obtained in U2-OS cell lines (Fig. 5A and B), which strongly indicated that miR-194 had an important role in reducing the migration and invasion of osteosarcoma *in vitro*.

The wound healing assay showed that cells in OE groups exhibited a significant decrease in migration rate compared to the other three groups. KD groups of SOSP-9607 cells (or U2-OS) nearly closed the wound 48 h after incubation, but not the other three groups (Fig. 5C and D).

### miR-194 inhibits tumor growth and metastasis of osteosarcoma in vivo

**Figure 6:**
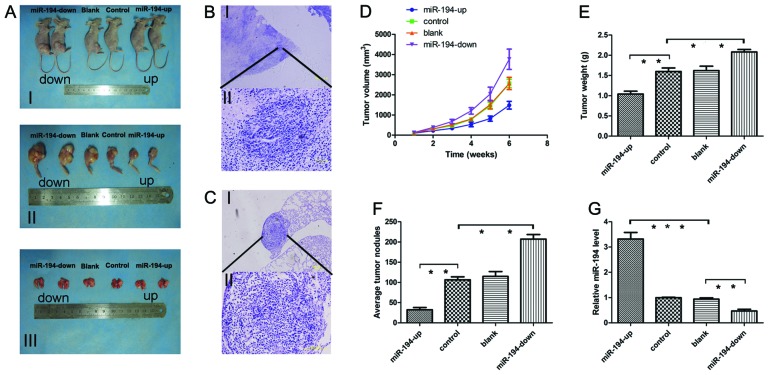
miR-194 inhibits tumor growth and metastasis of osteosarcoma *in vivo*. (A) I, Representative photographs of tumors on the right legs of mouse; II, Representative images of orthotopic tumors harvested 42 days after inoculation. III, Representative images of mouse lungs, 42 days post-inoculation. (B) Representative images of H&E-stained spontaneous orthotopic tumors at a magnification of ×40 (I) and ×400 (II). (C) Representative images of H&E-stained spontaneous lung metastases at a magnification of ×100 (I) and ×400 (II). (D) Tumor growth curves measured after the inoculation. The length (L) and width (W) of tumor were measured every 7 days after inoculation and the volume of tumor was calculated. (E) Orthotopic tumor weights 42 days post-inoculation. Data are presented as means ± SD. (F) Graph displaying the total number of tumor nodules per lung in four groups. Data are presented as means ± SD. (G) Forty-two days after inoculation, miR-194 expression levels were determined in orthotopic tumors. Columns, mean of three independent experiments; bars, SD; ^**^P<0.01, ^***^P<0.001.

Four groups of stable cells (OE, control, blank and KD SOSP-9607) were injected into proximal tibia of young nude mice. To evaluate tumor growth, the length (L) and width (W) of orthotopic tumor were measured every 7 days post-inoculation. The volume of tumor was calculated according to the formula: Volume = 1/2 × L × W^2^, and the growth curve of orthotopic tumor obtained. Progressive solid tumors were seen in all mice. By contrast, cells in OE groups produced much smaller tumors, while KD group generated the biggest size (Fig. 6A and D; P<0.05). The mice were sacrificed 42 days post-inoculation. The mean tumor weight ± SD of orthotopic tumors were as follows: OE group 1.04±0.159 g, control group 1.598±0.198 g, blank group 1.622±0.240 g and KD group 2.082±0.134 g (Fig. 6A and E). No significant difference was observed between the blank and control cells (p=0.842). The number of metastatic nodes was dramatically reduced in the nude mice in OE group compared with the other groups (Fig. 6A and F). Tumor and metastases were confirmed based on histopathological evaluation (Fig. 6B and C). Orthotopic tumors in the OE group expressed higher miR-194 levels compared with other groups (Fig. 6G) indicating that exogenous miR-194 significantly inhibited the tumor growth and metastasis *in vivo*.

### Expression of miR-194 in osteosarcoma and corresponding non-cancerous tissues

**Figure 7:**
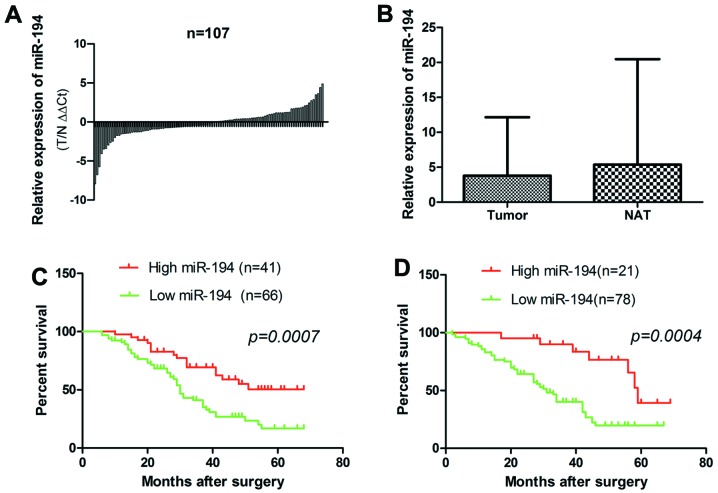
Expression level of miR-194 in 107 osteosarcoma patients and 99 osteosarcoma formalin- or paraformalin-fixed, paraffin-embedded (FFPE) tissues. (A) Relative levels of miR-194 in 107 surgical specimens of osteosarcoma and matched adjacent non-cancerous tissues was quantified by qRT-PCR. Data are presented as log2 fold change (ΔΔC_T_ values, tumor/non-cancerous tissues, T/N). (B) Means of miR-194 relative levels for 107 surgical specimens of osteosarcoma and the matched adjacent non-cancerous tissues. Data are presented as 2^-ΔΔCt^ values (p=0.291). (C) Decreased expression of miR-194 was correlated with poor survival in osteosarcoma patients. Log-rank tests show that patients with high miR-194 expression survived significantly longer (p=0.0007) than those with low miR-194 expression. The median miR-194 expression level (3.647) in the tumor samples was chosen as the cut-off point. (D) Decreased expression of miR-194 was correlated with poor survival in osteosarcoma FFPE patients. Log-rank tests show that patients with high miR-194 expression survived significantly longer (p=0.0004) than those with low miR-194 expression. The median miR-194 expression level (5.74) in the tumor samples was chosen as the cut-off point. Columns, mean of three independent experiments; bars, SD.

miR-194 expression was decreased in 59 of 107 (55.14%) tumor samples compared with their non-malignant counterparts by real-time PCR (Fig. 7A). U6 was used as a control. However, no statistically significant difference was observed between the cancer tissues (mean ± SD, 3.6467±7.44944) and matched non-tumor adjacent tissues (NATs) (mean ± SD, 5.3679±15.09357) (p=0.291) (Fig. 7B).

### Downregulation of miR-194 is associated with advanced clinicopathological features of osteosarcoma

The median miR-194 expression level in 107 patients with osteosarcoma was 3.647. Patients were divided into two groups according to their expression levels of miR-194, using its median as a cut-off: high miR-194 expression group (n=41) and low miR-194 expression group (n=66). As shown in Table I, we found statistically significant relationships between miR-194 expression and age (p=0.0015), clinical stage (p=0.019), distant metastasis (p=0.0251) and patient mortality (p=0.0065). No significant difference was observed between the expression of miR-194 and the patient gender (p=0.4038) and tumor size (p=0.6264).

The median of miR-194 expression levels in all 99 paraformalin-fixed, paraffin-embedded (FFPE) tissues with osteosarcoma was 5.74. The FFPE tissues were also divided: high miR-194 expression group (n=21) and low miR-194 expression group (n=78). As shown in Table II, we found statistically significant relationships between miR-194 expression and age (p=0.037), tumor size (p=0.041), clinical stage (p=0.039), distant metastasis (p=0.044) and patient mortality (p=0.013). No significant difference was observed between the expression of miR-194 and the patient gender (p=0.749). These results revealed that loss of miR-194 was associated with some clinicopathological features of OS.

### Downregulation of miR-194 confers poor prognosis in patients with osteosarcoma

Patients with high miR-194 expression survived significantly longer compared with low miR-194 expression based on the log rank test (Fig. 7C; p=0.0007). Similar results were obtained with FFPE tissues (Fig. 7D; p=0.0004). These results revealed that downregulation of miR-194 was associated with poor prognosis of OS.

To identify whether miR-194 was an independent prognostic covariate for osteosarcoma, we performed a multivariate Cox proportional hazards analysis. In the final multivariate Cox regression model, low levels of miR-194 expression in osteosarcoma (p=0.001, relative risk = 0.390) and distant metastasis (p=0.001, relative risk =2.386) were associated with a poor prognosis in terms of overall survival, independent of other clinical covariates (Table III). Similar results were obtained in FFPE tissues (Table III; p=0.023, relative risk = 0.371).

### CDH2 and IGF1R are potential targets of miR-194

**Figure 8:**
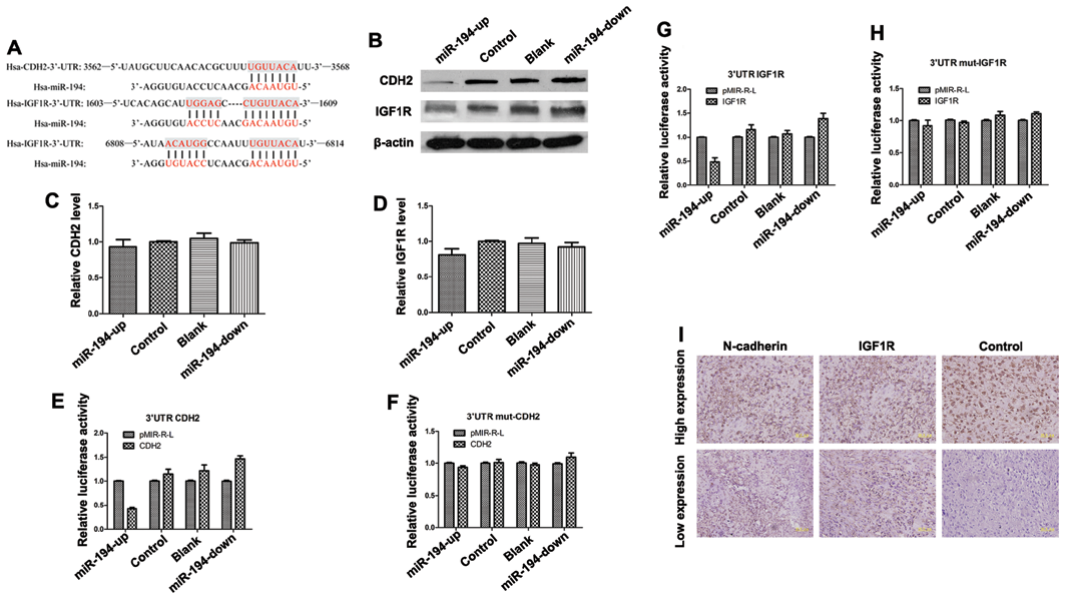
miR-194 target CDH2 and IGF1R in osteosarcoma. (A) Sites of complementarity sequences between microRNAs and CDH2or IGF1R mRNA. (B) Western blotting showed that miR-194 interacted with CDH2 or IGF1R and negatively regulated its expression at the translational level. (C and D) Real-time PCR revealed that miR-194 had no effect on CDH2 or IGF1R in mRNA level. (E and F) Luciferase assays indicated that miR-194 downregulated the expression of CDH2. Relative repression of the firefly Luciferase expression was standardized for transfection control, *Renilla* luciferase. PMIR-REPORT™ luciferase (pMIR-R-L; Promega) was used as empty vector. (G and H) Luciferase assays indicated that miR-194 downregulated the expression of IGF1R. Relative repression of the firefly Luciferase expression was standardized for transfection control, *Renilla* luciferase. PMIR-REPORT™ luciferase (pMIR-R-L; Promega) was used as empty vector. (I) Inverse correlation of expression of miR-194 and N-cadherin and IGF1R in human osteosarcoma. Expressions of N-cadherin and IGF1R were analyzed in 99 paraffin specimens of osteosarcoma tissues with immunohistochemical staining and classified as follows: 0–4, low; 5–9, high. All experiments were repeated three times in triplicate. Columns, mean of three independent experiments; bars, SD; ^*^P<0.05, ^**^P<0.01, ^***^P<0.001.

We examined the potential targets of miR-194 by searching the PicTar miRanda and TargetScan databases. We identified a conserved domain within the 3′-UTR of *CDH2 (N-cadherin)* and *IGF1R* with a potential miR-194 binding site (Fig. 8A). We examined the expression of *CDH2* and *IGF1R* on mRNA and protein levels in OE, blank, control and KD SOSP-9607 cells using real-time PCR and western blot analysis. The results showed that miR-194 had no effect on *CDH2* and *IGF1R* mRNA levels (Fig. 8C and D). However, the level of endogenous *N-cadherin* protein in OE SOSP-9607 cells was reduced compared with the other three cells normalized to an endogenous reference β-actin protein (Fig. 8B). Overexpression of *N-cadherin* protein was also found in KD SOSP-9607 cells compared with the other three groups of cells (Fig. 8B). Western blot analysis demonstrated a significant decrease in endogenous *IGF1R* levels in OE group compared with the other three groups (Fig. 8B). The results indicate that miR-194 may target *CDH2* and *IGF1R*.

We assessed the interaction of miR-194 with luciferase reporter assay in SOSP-9607 cells using a pMIR-REPORT™ Luciferase vector containing the 3′-UTR of *CDH2* or a control pMIR-REPORT™ Luciferase vector containing the same 3′-UTR with mutated miR-194 seed nucleotides. *Renilla* luciferase vector was used for normalization. The miR-194-up cells significantly repressed the luciferase activity of the vector with the wild-type *CDH2* 3′-UTR, whereas mutation of the seed sequence abolished this repression (Fig. 8E and F). Similar results of the *IGF1R* were also obtained (Fig. 8G and H).

### Expression of IGF1R and N-cadherin proteins were both inversely correlated with miR-194 expression and regulated the migration and invasion of osteosarcoma cells

We examined *IGF1R* and *N-cadherin* protein expression in 107 patients with osteosarcoma using real-time quantitative PCR. Of the 41 osteosarcoma cases with elevated miR-194, 25 (61.0%) showed low levels of *N-cadherin*. High levels of *N-cadherin* were present in 42 of 66 (63.6%) cases with downregulated miR-194 (p<0.05). Of the 41 osteosarcoma cases with elevated miR-194, 29 (70.7%) had low levels of *IGF1R*. High levels of *IGF1R* were seen in 47 of 66 (71.2%) cases with downregulated miR-194 (p<0.001) (Table IV). These findings suggest that expression of the *IGF1R* and *N-cadherin* proteins were inversely correlated with miR-194 expression in osteosarcoma.

We then examined *IGF1R* and *N-cadherin* protein expression in 99 paraffin specimens of osteosarcoma using immunohistochemistry analysis. Representative images of *IGF1R* and *N-cadherin* are shown (Fig. 8I) and analyzed (Table IV). Of the 21 osteosarcoma cases with elevated miR-194, 16 (76.2%) showed low levels of *N-cadherin*. High levels of *N-cadherin* were present in 51 of 78 (65.4%) cases with downregulated miR-194 (p<0.01). Of the 21 osteosarcoma cases with elevated miR-194, 15 (71.4%) had low levels of IGF1R. High levels of *IGF1R* were seen in 62 of 78 (79.5%) cases with downregulated miR-194 (p<0.001). These findings suggest that expression of the *IGF1R* and *N-cadherin* proteins were inversely correlated with miR-194 expression in osteosarcoma.

The expression of *N-cadherin* was associated with clinical stage (p=0.0354), distant metastasis (p=0.0271) and survival (p=0.0014), while the expression of *IGF1R* was associated with tumor size (p=0.0101), distant metastasis (p=0.0259) and survival (p=0.0253) (Table I).

## Discussion

Osteosarcoma is the most frequent primary solid malignancy of bone, which is defined by the presence of malignant mesenchymal cells which produce osteoid and/or immature bone (40). Approximately 20% of patients present with lung metastases at initial diagnosis and in 40% of patients metastases occur at a later stage. Eighty percent of all metastases arise in the lungs, most commonly in the periphery of the lungs, and exhibit resistance to conventional chemotherapy (41).

Uncontrolled cell proliferation and aggressive tumor cell metastasis are two essential steps during cancer progression. Therefore, in the present study, we investigated the effects of miR-194 on tumor growth and metastasis of osteosarcoma. We showed that overexpressed miR-194 significantly suppressed proliferation, migration and invasion of SOSP-9607 and U2-OS cells *in vitro*. Our mouse model showed that miR-194 also significantly inhibited orthotopic tumor growth and lung metastasis *in vivo*. We have performed the largest study to date that assessed the expression levels of miR-194 in osteosarcoma by real-time PCR. However, no significant difference of miR-194 expression was found in 107 cancerous and adjacent non-cancerous tissue pairs. Tissue specificity might be one of the reasons which was associated with the differences in miR-194 expression, as observed in cancer miRNA signatures across different organs (42). Furthermore, different osteosarcoma cell lines show different expression profiles of invasion, motility and colony formation, with different mRNAs and miRNA expression (43). We observed that decreased expression of miR-194 was correlated with cancer progression and poor prognosis in osteosarcoma patients, independent of other clinicopathologic factors. Therefore, upregulated miR-194 was very effective in inhibiting tumor growth and metastasis indicating that miR-194 functions as a tumor suppressor gene and as a potential therapeutic target.

Generally, metastatic models are conducted in nude mice by injecting human osteosarcoma cells either intravenously or subcutaneously (32). However, these models are not clinically relevant since osteosarcoma does not occur spontaneously (44). In the present study, we selected a spontaneous metastatic model involving orthotopic transplantation of osteosarcoma cells resulting in spontaneous pulmonary metastases (32). The microenvironment of tibia in nude mice resembled the tumor progression and metastases development clinically.

Cadherins have a role in Ca^2+^-dependent cell-cell interaction (45) as well as acting as metastasis promoting or suppressing proteins in different cancers (46,47). Insulin and insulin-like growth factor receptor (IGFR)-mediated molecular pathways are important effectors of neoplastic transformation in non-small cell lung cancer (48) and squamous cell carcinoma of the head and neck (49). *IGFIR* has a major role in cancer cell proliferation and survival, and confers resistance to cytotoxic, hormonal and targeted therapies (50). Our results indicate that miR-194 interacted with *N-cadherin* and *IGF-IR* and negatively regulated their expression at the translational level, which also indicated that miR-194 may suppress tumor growth and metastasis in osteosarcoma cells by down-regulating *N-cadherin* and *IGF-IR*.

Unlike siRNAs, which silence the expression of a single gene, miRNAs mainly silence the expression of multiple genes simultaneously. It is estimated that an average miRNA may have more than 100 targets (51). It is crucial to identify additional target genes that mediate the miR-194-induced regulation of tumor metastasis. Predicting and identifying the miR-194-targeting genes provides an experimental basis for further research on regulatory mechanism of miR-194. By using TargetScan 5.1 and PicTar, we predicted putative genes of miR-194, and obtained several putative targets correlating with tumor growth or metastasis, such as *QKI*, *KIAA1239*, *EPHA5*, *NACC2*, *MCTS1* and *SAMD4A*. In general, the discovery of miRNA and their functions, has introduced a new dimension to our existing knowledge of signaling molecules and pathways for more precise therapeutic targeting. Further investigation is required for characterization of miR-194 and other miRNAs as prognostic and/or diagnostic markers in human osteosarcoma.

In conclusion, the results demonstrate that miR-194 affected the growth and metastasis of osteosarcoma cells both *in vitro* and *in vivo*. Overexpression of miR-194 downregulated the expression of N-cadherin and IGF-IR protein, suggesting that miR-194 functions as tumor suppressor probably by downregulating N-cadherin and IGF-IR in osteosarcoma. Downregulation of miR-194 may be associated with tumor aggressiveness and tumor metastasis of osteosarcoma, suggesting that miR-194 may be an independent prognostic marker for osteosarcoma. Other putative miR-194 target genes that are potentially associated with the growth and metastasis of osteosarcoma cells should be investigated. Finally, miR-194 may prove to be a promising gene therapeutic agent. It could be informative to confirm the putative target genes and further investigate the underlying molecular mechanisms of miR-194 as a tumor suppressor gene in osteosarcoma.

## Figures and Tables

**Table I:** Relationship between expression of miR-194, N-cadherin and IGF1R and clinicopathological factors in 107 osteosarcoma patients.

		miR-194 expression			N-cadherin expression			IGF1R expression		
							
Characteristics	No.	Low no.	High no.	p-value	Low no.	High no.	p-value	Low no.	High no.	p-value
Gender				0.130			0.8110			0.4753
Male	62	42	20		29	33		26	36	
Female	45	24	21		20	25		22	23	
Age (years)				0.0052^a^			0.0957			0.5904
≥18	35	15	20		12	23		17	18	
<18	72	51	21		37	35		31	41	
Tumor size (cm^2^)				0.1098			0.0769			0.0172^a^
≥50	47	25	22		17	30		15	32	
<50	60	41	19		32	28		33	27	
Clinical stage				0.0034^a^			0.0234^a^			0.1218
IIA	32	13	19		20	12		18	14	
IIB/III	75	53	22		29	46		30	45	
Distant metastasis				0.0058^a^			0.0081^a^			0.0139^a^
Yes	52	39	13		17	35		17	35	
No	55	27	28		32	23		31	24	
Status				0.0065^a^			0.0037^a^			0.0221^a^
Survival	45	21	24		28	17		26	19	
Death	62	45	17		21	41		22	40	

a Statistically significant p-values.

**Table II:** Relationship between expression of miR-194 and clinicopathological factors in 99 osteosarcoma formalin- or paraformalin-fixed, paraffin-embedded (FFPE) tissues.

	miR194 expression		
	
Characteristics	No. of cases	Mean ± SD	p-value
Gender			0.749
Male	55	5.9304±13.23676	
Female	44	4.6715±6.96565	
Age (years)			0.037^a^
≥18	41	11.5227±15.43802	
<18	54	1.4141±2.63859	
Tumor size (cm^2^)			0.041^a^
≥50	36	1.3000±3.38195	
<50	63	7.7371±12.95206	
Clinical stage			0.039^a^
IIA	27	12.9419±15.69269	
IIB/III	72	1.6235±4.33862	
Distant metastasis			0.044^a^
Yes	48	1.6254±4.66794	
No	51	10.5140±14.59265	
Status			0.013^a^
Survival	47	9.4371±13.60451	
Death	52	0.5538±0.58681	

a Statistically significant p-values.

**Table III:** Multivariate cox regression analysis of prognostic variables in osteosarcoma and osteosarcoma FFPE tissues.

	Variables	B	P-value	Wald	Relative risk	95% confidence interval
107 osteosarcoma tissues	miR-194 expression	−0.943	0.001^a^	11.813	0.390	0.228–0.667
	Age	−0.020	0.937	0.006	0.980	0.595–1.614
	Clinical stage	−0.261	0.297	0.771	0.771	0.431–1.378
	Distant metastasis	0.870	0.001^a^	10.493	2.386	1.410–4.038
	Tumor size (cm^2^)	0.470	0.091	2.851	1.599	0.927–2.759
99 FFPE tissues	miR-194 expression	−0.991	0.023^a^	5.193	0.371	0.158–0.871
	Age	0.036	0.893	0.018	1.037	0.612–1.756
	Clinical stage	−0.300	0.321	0.985	0.741	0.409–1.340
	Distant metastasis	0.898	0.005^a^	7.913	2.455	1.313–4.592
	Tumor size (cm^2^)	0.308	0.260	1.269	1.360	0.796–2.324

a Statistically significant p-values.

**Table IV:** Inverse correlation of expression of miR-194 and N-cadherin and IGF1R in osteosarcoma (using real-time quantitative PCR) and osteosarcoma FFPE tissues (using immunohistochemistry analysis).

	Group	High miR-194, n (%)	Low miR-194, n (%)	In all
107 osteosarcoma tissues	High N-cadherin	16 (39.0)	42 (63.6)	58
	Low N-cadherin	25 (61.0)	24 (36.4)	49
	In all	41	66	107
	High IGF1R	12 (29.3)	47 (71.2)	59
	Low IGF1R	29 (70.7)	19 (28.8)	48
	In all	41	66	107
99 FFPE tissues	High N-cadherin	5 (23.8)	51 (65.4)	56
	Low N-cadherin	16 (76.2)	27 (34.6)	43
	In all	21	78	99
	High IGF1R	6 (28.6)	62 (79.5)	68
	Low IGF1R	15 (71.4)	16 (20.5)	31
	In all	21	78	99
